# Imaging in Gender Affirmation Surgery

**DOI:** 10.1007/s11934-020-01029-3

**Published:** 2021-01-30

**Authors:** Omar Hassan, Derek Sun, Priyanka Jha

**Affiliations:** Department of Radiology, Abdominal Imaging and Ultrasound Section, University of California, San Francisco, 505 Parnassus Ave, box 0628, San Francisco, CA 94143-0628 USA

**Keywords:** Transgender imaging, Gender affirmation surgery, Bottom surgery, Phalloplasty complications, Metoidioplasty, Penoscrotal inversion vaginoplasty

## Abstract

**Purpose of Review:**

This review summarizes recent developments in gender affirmation surgery, imaging findings in patients undergoing these surgeries, focusing on common postoperative radiologic appearances, complications, and pitfalls in interpretation.

**Recent Findings:**

The imaging workup of masculinizing and feminizing genitourinary surgeries uses multiple modalities in presurgical planning and within the immediate and long-term postoperative period. CT and MRI can help identify immediate and remote postoperative complications. Fluoroscopic examinations can diagnose postoperative urethral complications after gender affirmation surgeries. Lastly, the patients can undergo imaging for unrelated acute and chronic pathology, and knowledge of these imaging findings can be very helpful.

**Summary:**

Imaging plays a significant role in the care of transgender patients and, particularly, in those pursuing gender affirmation surgery. As insurance coverage expands for these surgical procedures, radiologists should be prepared to encounter, understand, and interpret pre and postoperative findings.

## Introduction

Gender affirmation surgeries (GAS) include a variety of surgical procedures intended to support transgender individuals experiencing gender dysphoria, which can help align their physical sexual characteristics to their desired gender expression. Gender dysphoria includes conditions wherein the person’s self-perceived gender is not concordant with their biological sex, which results in discomfort and mental angst [1]. While other descriptors have been used in the past to describe these surgeries, using the GAS terminology replaces more pejorative and less compassionate terms. It is estimated that in the USA, nearly 1.6 million Americans, or 1 in 250 people, self-identify as transgender. While many of these people choose to align with their desired gender through altering their clothing and cosmetic appearance, a smaller fraction may opt to pursue GAS [2, 3]. Among this group, transgender women are significantly more likely to pursue surgical options over transgender men [4••]. As a result of broadening insurance coverage for therapies to treat gender dysphoria over the last decade, many patients who undergo GAS are increasingly likely to present in non-specialized clinics and emergency departments across the country [5]. Consequently, an understanding of the social, medical, and surgical issues unique to this patient population is imperative for healthcare professionals to provide complete and compassionate care [1]. Increasingly, this includes radiologists and urologists not routinely involved in the care of the transgender patient.

Broadly speaking, gender affirmation surgery is categorized into facial feminization or masculinization and chest reconstruction, otherwise collectively known as “top surgery,” and surgical procedures involving the genitalia [6]. A summary of these procedures is provided in Table 1. Our discussion will focus on masculinizing and feminizing GAS, colloquially referred to as “bottom surgery,” with emphasis on immediate and late expected postoperative imaging appearances and recognizing immediate and remote complications. Imaging plays a crucial role in the care of patients pursuing gender affirmation surgery and using multiple imaging modalities is especially important to address specific questions from preoperative planning to post-surgical evaluation.

**Table 1:** Gender-affirming surgical options. Transgender individuals may pursue none, one, or combinations of the described procedures

Transmasculine surgery	
Thyroplasty	
Subcutaneous mastectomy	
Hysterectomy	
Unilateral or bilateral oophorectomy	
Vaginectomy	
Metoidioplasty, phalloplasty, scrotoplasty	
Implantation of testicular prostheses or erectile devices	
Transfeminine surgery	
Facial feminization	
Thyroid chondroplasty	
Glottoplasty (voice surgery)	
Breast augmentation	
Penectomy, orchiectomy	
Vaginoplasty, labiaplasty, clitoroplasty	

## Masculinizing Gender Affirmation Surgery

### Goals and Techniques

Transmasculine patients may choose to pursue GAS to fulfill the needs for standing micturition and penetrative intercourse. Hence, the goal of masculinizing surgery is to remove female external genitalia (vaginectomy) and internal reproductive organs (hysterectomy and oophorectomy), creating an aesthetically acceptable neophallus (phalloplasty) that achieves standing micturition as well as adequate bulk and sensation for penetrative intercourse [7]. Because many of the surgical procedures are performed in several stages, patients may choose which components of the surgery they wish to have performed. For example, some patients may choose to undergo phalloplasty while retaining their uterus and ovaries. Patients are counseled regarding their options and the final decision is based on patient preference as well as anatomic considerations. Metoidioplasty and phalloplasty are the two most common approaches to transmasculine genital affirmation surgeries.

### Metoidioplasty

Metoidioplasty is a procedure using clitoral hypertrophy and clitoral release to form masculine-appearing external genitalia. This is a simpler, lower-complication procedure that may maintain adequate sexual sensation, however with reduced bulk of the neophallus. Advanced urethral reconstructive techniques may allow for standing micturition as well. Metoidioplasty is often preceded by at least 1 year of hormonal supplements to stimulate hypertrophy of the clitoral tissue and maximize the bulk of the phallus construct. In a simple metoidioplasty, the urethral plate is freed from its attachments and lengthened [8]. If standing micturition is desired, the urethra may be lengthened with the use of vulvar mucosal tissue from the anterior vaginal wall, a procedure which is similar to the repair of congenital hypospadias. Occasionally, buccal mucosa or vascularized local skin grafts can be used to form the distal aspect of the urethra for desired urethral elongation [9••].

### Phalloplasty

Phalloplasty includes the creation of a neophallus using muscular flaps [7]. This procedure creates a more aesthetically pleasing neophallus with penetrative bulk while achieving standing micturition. However, the procedure is more intensive, may require a two-stage approach, and is associated with more complications. Compared to metoidioplasty, there is decreased sexual sensation. In addition to phalloplasty, patients may also undergo vaginectomy, laparoscopic total abdominal hysterectomy, salpingo-oophorectomy, and lastly, placement of penile and testicular prostheses, although some patients may not elect to pursue all of these components [10].

**Fig. 1:**
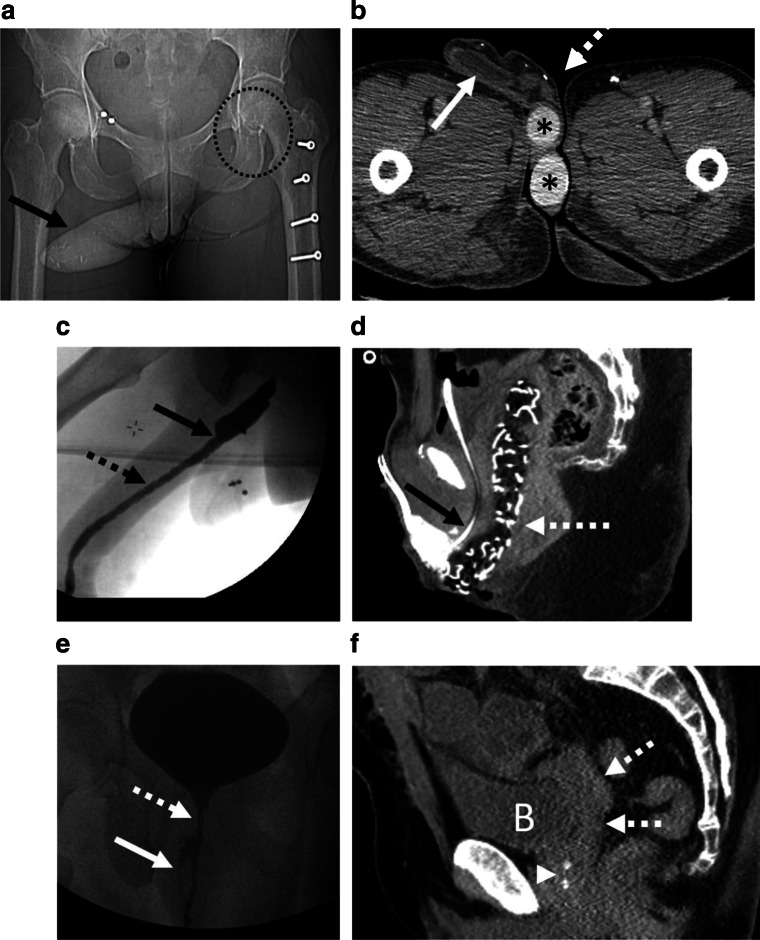
**a** Scout view from a CT in a 49-year-old transmasculine patient demonstrates the neophallus (arrow) created by anterolateral thigh pedicled flap. Surgical clips in the left groin (circle) demonstrate the site of mobilization of the vascularized pedicle. The left side is the preferred side for harvesting the muscular flap due to higher takeoff of left common femoral artery and a larger number of perforating branches. **b** Axial contrast-enhanced CT in the same patient demonstrates the neophallus. Inside the neophallus, the neourethra (dotted arrow) is visualized created using the “tube within the tube” technique. Alongside the urethra, a single barrel inflatable penile prosthesis (white arrow) is seen. Testicular prostheses (*) are present as well. **c** Fluoroscopic retrograde urethrogram in a 38-year-old transmasculine patient status post phalloplasty demonstrates an abrupt change in urethral caliber at the junction of the native urethra and neourethral pars fixa (dotted arrow), suggestive of a stricture (solid black arrow). The neourethra (dotted arrow) which was created using labia minora can normally have a diffusely irregular contour, and should not be misinterpreted as stricturing if there is no change in caliber. Urodynamic studies can be obtained to further confirm radiographic findings. **d** Sagittal contrast CT obtained in the immediate postoperative period in a 29-year-old transfeminine patient status post penoscrotal inversion vaginoplasty demonstrates the neovagina (dotted arrow) filled with packing material. A Foley catheter (black arrow) is present in the urethra status post urethral shortening. **e** Voiding cystourethrogram performed in a 32-year-old transfeminine patient status post penoscrotal inversion vaginoplasty and now complaining of urine dribbling from the neovagina demonstrates the urethra (dotted arrow) opacified with contrast. In addition, a parallel track of contrast is seen consistent with a fistulous connection between the urethra and the neovagina. **f** 48-year-old transfeminine patient with mature enteric vaginoplasty. The decompressed neovagina (dotted arrow) is seen behind the bladder (B). Inferior to the bladder and anterior to the neovagina, the atrophied prostate (arrowhead) is present and contains a few coarse calcifications. Prostate remains in situ in transfeminine genital surgeries and should not be misinterpreted as a soft tissue mass

Muscular flaps used for neophallus creation can be obtained as a pedicled flap from anterolateral thigh (ALT) or as free flaps from the forearm (radial forearm free flap, RFFF) (Fig. 1a) [7, 11]. Neourethra can be created using forearm skin flaps or vaginal or buccal mucosa in a “tube within a tube” fashion [12]. This neophallic urethra, sometimes referred to as the pars pendula, is anastomosed to the lengthened perineal urethra, sometimes referred to as the pars fixa. If standing micturition is not a priority for the patient, a simpler perineal urostomy can be created. Nervous coaptation connects the cutaneous nerves of the forearm to the ilioinguinal or clitoral nerves. Each donor site is covered with a skin graft.

The benefits of the radial forearm free flap approach include the highest chance of successful neourethra and urinary conduit creation while using relatively hairless ulnar skin for creating a penile urethra [7]. However, for thinner patients, there may not be adequate muscular bulk for penetrative intercourse. Additionally, since this is a free flap, arterial and venous vascular anastomoses are required. The rotated anterolateral thigh flap, freed from its attachments and tunneled underneath the sartorius and rectus femoris muscles, avoids an arteriovenous anastomosis and neural coaptation, but allows for better bulk compared to the radial forearm free flap construct [7]. The hairier skin of the thigh can lead to urinary issues over a long term, even if electrocauterization of the hair follicles has been performed.

If scrotoplasty is desired, tissue from the labia majora is used, sometimes preceded in the initial stage with labia majora spacer placement to expand the skin for the future neoscrotum creation. Depending on patient preference, penile or testicular prostheses can be implanted to create a more functional and realistic end result (Fig. 1b) [10, 13, 14]. Vaginectomy with or without hysterectomy can be performed via cauterization or resection of the vaginal cavity, followed by suture closure of the peritoneum and paravaginal space.

### Complications of Masculinizing Surgery

Overall, metoidioplasty is a significantly less invasive and lower-risk procedure than phalloplasty. Immediate postoperative complications of metoidioplasty are those expected for typical surgical procedures: hematoma, superficial or deep site infection, abscess, or graft failure. These are typically diagnosed via computed tomography (CT) and will be discussed in the following section. Complications more specific to metoidioplasty include urethral complications, which can present in the immediate postoperative period to several years from surgery, with the mean time to stricture or fistula formation being reported at 3 months in certain studies [12]. Anastomotic stricture, urethral stenosis, diverticula or sinuses, and leak are common urologic complications and are typically diagnosed via fluoroscopic examination, detailed below.

Phalloplasty is significantly more fraught with complications and the risks inherent to phalloplasty increase as more procedures or stages are added [7]. Like metoidioplasty, common immediate postoperative complications are seen, including seroma, hematoma, abscess, superficial or deep site infection, and graft failure. However, given the involvement of a rotational or free flap, arterial or venous thrombosis or bleeding is a very distinct possibility. The rate of graft failure is higher and is typically diagnosed via clinical exam. Vaginectomy after metoidioplasty or phalloplasty can be complicated by mucocele if there retained vaginal tissue, and this is a particular concern if only electrocauterization has been performed [15, 16]. However, overdissection during vaginectomy raises the risk of rectal or bladder injury and hence, an optimal balance must be struck by the surgeon. Fistulization is another complication that typically presents clinically and is diagnosed with pelvic CT or MRI. Phalloplasty is also notoriously bogged by urethral complications given the length of the neourethra and the two anastomoses at the native urethra-pars fixa junction and the pars fixa-pars pendulans junction [4••, 5, 6]. In most of the cases, urethral complications resolve with diversion and conservative management. Leaks or fistulae may be watched for several months for spontaneous resolution before a decision is made to re-operate. Urethral strictures can occur at any time but typically arise 6 to 18 months postoperatively [17]. These can be endoscopically dilated or, if recurrent, re-operated with a one- or two-stage repair using a buccal graft [18•]. Finally, if penile or testicular prostheses are employed, common complications include infection, implant failure, erosion through soft tissue, and implant migration [19]. For unclear reasons, the rate of prosthesis failure is higher in postoperative transgender patients compared to cis-gender individuals requiring prosthesis [20, 21].

### Imaging Considerations

Generally, preoperative imaging is not necessary for metoidioplasty. However, for patients desiring phalloplasty, certain tests are obtained depending on the surgical approach. For RFFF, an Allen or Barbeau test is initially performed. Equivocal results from these tests or when patients have previously undergone surgery for their arms or forearms may prompt evaluation with CT angiography and sometimes, catheter-directed angiography by interventional radiology. Preoperative workup for ALT flaps involves bilateral lower extremity CT angiograms to estimate which leg has the superior most origin of the lateral femoral cutaneous artery (LFCA), which leg has the greatest number of LFCA perforators, and overall bilateral subcutaneous depth to estimate construct size. Usually, the left side is preferred due to a more superior takeoff of the LFCA, which facilitates graft rotation and positioning, while the increased number of perforator branches increases the likelihood of graft perfusion and hence success [9••].

The mainstay imaging modality for immediate postoperative complications of metoidioplasty or phalloplasty is CT. Routine contrast-enhanced examinations of the abdomen and pelvis are typically sufficient to diagnose usual postoperative hematomas, seromas, and abscesses. Hematomas will appear as intermediate density collections, about 30–60 Hounsfield units, usually without rim enhancement unless infected or longstanding, in which case a fibrous rim develops [9••]. If there is clinically suspected arterial bleeding in the setting of hemorrhage, CT angiography can be employed. CT angiography can be useful for suspected arterial or venous thrombosis as well, with delayed phases facilitating the diagnosis of venous thrombosis [9••].

When a collection persists and there is diagnostic uncertainty about its nature, a pelvic MRI with fat saturation can be obtained. In these instances, the fat-saturated sequence can help distinguish between fat necrosis and subacute hematomas, both of which typically appear T1 hyperintense. Sometimes, ill-defined persistent densities near the surgical site are seen on CT; these can typically be ascribed to granulation tissue [22••].

While mucocele from retained vaginal mucosa is the most common complication of vaginectomy, complications related to rectal and bladder injury from overdissection of the paravaginal space pose significant challenges for clinical management. With an incidence of 4–5%, these complications are frequently identified and repaired intraoperatively but may present in the subacute to late postoperative period with fistula formation, infection, and abscesses. Clinicians should use real-time fluoroscopic examination with a water-soluble contrast agent, including enemas or fistulograms for rectovaginal fistulae and cystography for vesicovaginal or urethrovaginal fistulae [9••]. Pelvic MRI with a perianal fistula protocol can provide additional information for surgical planning by displaying the entire length of the fistula/sinus tracts and can help with diagnosis when fluoroscopy fails to detect an abnormality. Occasionally, fistulae can arise between the neourethra and the vaginectomy site, which can be clinically diagnosed and closed surgically [17]. More insidiously, internal fistulas can develop and present as recurrent urinary tract infections, urosepsis, urinomas, or urine per rectum. In this instance, contrast-enhanced pelvic MRI is the gold standard for diagnosis [22••].

Neourethral complications constitute the most common and challenging complications of phalloplasty. Because of the length of the neourethral construct, two anastomotic sites, and its relative flaccidity, postoperative phalloplasties suffer from urethral strictures, stenosis, fistulas, sinuses, and diverticula [17]. Patients may present at any point in their postoperative course up to several years later with a secondary stream, dribbling, acute urinary retention, infection, sepsis, or renal failure. Due to the altered anatomy, Foley catheter placement is not straightforward and may require endoscopic guidance with consideration for the placement of a suprapubic catheter instead. The mainstay for diagnosis of neourethral abnormalities is fluoroscopic retrograde urethrography (RUG) or voiding cystourethrography (VCUG). VCUG serves as the workhorse modality to detect abnormalities and RUG serves as the confirmatory exam. At some institutions, both these tests can be performed concurrently. Depending on the institutional practice, these exams may be performed by either urologists or radiologists and can help detect strictures and fistula. However, a stricture can be overdiagnosed on fluoroscopic studies and may require urodynamic studies to determine whether detected areas of narrowing are clinically significant (Fig. 1c) [17]. Three locations are implicated in the vast majority of urethral complications: the native urethra-pars fixa anastomosis, pars fixa-pars pendulans anastomosis, and the ventral pars pendulans, which has an entire suture line running along its course as part of the “tube-within-a-tube” neophallus construct and strictures can develop anywhere along this course. At the posterior pars pendulans are bilateral outpouchings termed “horns” of the pendulans, which are normal postoperative findings. One imaging pitfall, however, is mistaking the horns of the pendulans for urethral diverticula or sinuses. The distinction involves the shape of the horns: pendulans horns are relatively triangular and symmetric, while pathologic abnormalities are rounded or linear and usually unilateral [18•, 23].

Finally, complications can arise from the testicular or penile prosthetics implanted in transgender patients. Implant failure is typically diagnosed clinically, while implant erosion through soft tissue, infection, or migration is typically diagnosed on CT.

Because of the various combinations of masculinizing “bottom” surgery, the interpreting radiologist should remain open to all considerations with all radiographic findings. This is especially true when a history of gender affirmation surgery is withheld or not known by the primary team. Unexpected or incongruent genital imaging on CT may prompt clarification. Operative reports when accessible can be immensely helpful. Even then, the specific surgical procedures may be unclear. Because patients may choose to pursue variable components of the procedure and can sometimes elect to forgo hysterectomy with or without salpingo-oophorectomy, a uterus can be present and should not be confused for a pelvic mass. The uterus can develop myomas as in cis-women and can raise alarm for an ominous mass, especially when a neophallus is present. Additionally, some patients may undergo only a unilateral salpingo-oophorectomy or bilateral salpingectomy with unilateral oophorectomy for hormonal reasons. Consequently, the remaining ovary may be confused for a solid-cystic pelvic mass as well. The interpreting radiologist should also remain alert of the possibility of obstruction and fistula. Evidence of testicular or penile prosthetic infection or migration should also be evaluated [3, 14, 20].

## Feminizing Gender Affirmation Surgery

### Goals and Techniques

Transfeminine patients may choose to pursue GAS to fulfill their desire of aligning their external genitalia with their female gender as well as for receptive intercourse. The modern surgical approach to feminizing genital surgery is the penoscrotal inversion vaginoplasty (PIV), although second-line and older alternatives including enteric vaginoplasty are also occasionally performed. Components of transfeminine surgery include penectomy, orchiectomy and penoscrotal inversion vaginoplasty, or increasingly less commonly, enteric vaginoplasty [24].

### Penoscrotal Inversion Vaginoplasty

This technique uses the genital skin to create a neovagina and vulva for patients assigned male sex at birth [25]. Briefly, the perineum, pelvic floor muscles, and rectoprostatic space are dissected to create a space for a vaginal vault of appropriate depth and angle of inclination. The penis is degloved and the skin is inverted to create the lining of the new vaginal vault. The corpora cavernosa are resected while the glans is refashioned into a neoclitoris [26]. The urethra is shortened and the meatus is repositioned to create a urethral opening on the perineal surface. Finally, bilateral or unilateral orchiectomy can be performed and the scrotal skin is used to create the neolabia [25]. The vagina is packed in the immediate postoperative period and patients are instructed to use serial dilators for the rest of their lives to maintain neovaginal patency for receptive intercourse. In certain cases, such as prepubertal hormone blockage or with extended hormonal suppression, there may be limited penoscrotal skin available to allow for adequate vaginal depth and this may necessitate the use of grafts such as peritoneal augmentation [27, 28].

The functional success of PIV is based on the overall dimensions of the neovagina as well as its angle of inclination. Postoperatively, the neovagina should be at least 8 cm in length with an angle of inclination around 54° to allow for receptive intercourse. In comparison, cis-female adult vaginas measure an average depth of 6–13.5 cm with an angle of inclination of approximately 40°. The minimum thickness of the rectovaginal septum, defined as the total width of the posterior wall of the neovagina and anterior wall of the rectum, should be at least 3 mm. The surgical technique is hence important to balance the amount of penile skin available, the patient’s pelvic dimensions, and optimal dissection of the rectoprostatic space, taking caution to maintain an adequate rectovaginal septum, and to avoid rectal injury [4••]. While sufficient separation is necessary between the prostate and the bladder anteriorly and the rectum posteriorly, overzealous dissection can lead to fistulous connections involving the neovaginal septum.

### Enteric Vaginoplasty

If a previous penoscrotal inversion vaginoplasty has failed or the skin is thought to be markedly deficient, intestinal interposition graft can be used for vaginoplasty. This approach is usually considered as a salvage option in current practice. Enteric vaginoplasty was first developed as an approach for vaginoplasty in transfeminine individuals desiring GAS. However, due to the risk of stenosis, excessive mucus production, and malodor, this has fallen out of favor in recent decades but is still used as a second-line alternative. The approach is also limited by the inherent risks of abdominal resection. A slightly less invasive alternative to augment vaginal depth uses a peritoneal flap, which boasts a significantly easier tissue transfer process and is self-lubricating [29]. However, this approach still requires abdominal surgery with its inherent risks [4••].

### Complications of Feminizing Gender Affirmation Surgery

As in most surgical procedures, hematoma, abscess, seromas, and surgical site infection are common complications of PIV [30–32]. Superficial wound dehiscence is a relatively common complication as well and treated conservatively. Meatal stenosis is common in patients who have undergone transfeminine GAS [17]. In an effort to create a neovagina of adequate length and depth, over dissection of the rectoprostatic space may lead to rectal or bladder injury with eventual rectovaginal, vesicovaginal, and urethrovaginal fistulae formation [30]. Over the longer-term, however, the procedure may fail due to poor patient compliance with lifelong dilator usage. Such patients may develop neovaginal stenosis or inadequate length over time, which in turn can precipitate paraneovaginal fluid collections or even urethral stenosis [32]. Pelvic floor prolapse may also develop [27, 28].

### Imaging Considerations

On CT, the neovagina appears as a tubular, soft tissue structure in the pelvis, with an opening at the perineum and a blind end in the pelvis. The neovagina is typically surrounded by granulation tissue or scar tissue, which is heterogeneous in density on CT and can be low to intermediate signal intensity on MRI. The perineal area where the glans was mobilized to create a sensate clitoris can appear hyperenhancing. Vaginal dilators can be seen on CT in the outpatient setting and care should be taken to avoid suggesting a retained foreign body or a tampon. Imaging evaluation includes measuring and reporting overall neovaginal depth and anterior-posterior width and the thickness of the rectovaginal septum (designated as the total width of both the neovaginal and rectal walls). The angle of inclination, measured as the angle of the neovagina with respect to a line drawn between the inferior pubic symphysis and coccyx (pelvic outlet), should also be reported. On average, the angle of inclination in postoperative feminizing surgery has been reported at 54°, while in cis-women, it measures roughly 40° [4••].

In the immediate postoperative period, vaginal packing material is generally expected as a bundle or nest of radiodense linear material with surrounding air. Care should be taken not to misinterpret these as unintentionally retained foreign objects, which may lead to their removal (Fig. 1d). A small amount of fluid in the immediate postoperative period is also to be expected and not misinterpreted as an abscess. Small peri-neovaginal hematomas are common [30]. As in masculinizing surgery, fat necrosis versus subacute hematoma can be difficult to distinguish on CT and even pelvic MRI as both have T1 hyperintense signal; however, fat-saturated sequences can be helpful to differentiate the two entities.

In cases of overzealous dissection of the rectoprostatic space, a fistulous connection can develop with the neovagina or a leak from the bladder or rectum, which can be evaluated fluoroscopically (Fig. 1e) [30]. Similar to transmasculine surgeries, radiographically occult disease can be evaluated with pelvic MRI, which allows for higher soft tissue contrast. T2-hyperintense, linear or branching structures between the neovagina and the bladder or rectum, and urethral-neovaginal fistulae can also be developed (Fig. 1e).

Residual erectile tissue is sometimes seen at the expected region of the base of the penis and should be reported by the interpreting radiologist. This can be a potential source of bleeding, and may even cause transient urinary retention by stenosing the neourethra. Additionally, engorged erectile tissue during sexual intercourse can be distressing for patients.

Neovaginal stenosis is a clinical diagnosis and can be seen on imaging as a fluid distended vagina. It can also lead to urethral stenosis if longstanding [17]. As in masculinizing surgery, fluoroscopic studies such as VCUG can help detect urethral abnormalities such as stenosis and fistulous connections. Urodynamic studies could distinguish between radiographically apparent stenoses versus clinically significant stenoses if multiple areas of abnormality are detected on fluoroscopic evaluation.

Because the pelvic floor muscles and perineum are dissected and neovagina is simply sutured into the paravaginal space, neovaginal prolapse can occur and is typically clinically evident. The degree of prolapse can be estimated on routine pelvic MRI with dynamic sequences. When prolapse is not present at rest, patients can be asked to Valsalva or recreate the motion that precipitates prolapse at the time of dynamic MRI. Additionally, given the broader field of view on MRI, associated bladder, urethral, or bowel prolapses can also be evaluated, aiding preoperative planning [22••, 23].

Lastly, all practitioners should be aware that the natal prostate remains present in transfeminine patients and presents an atrophic, small soft tissue density seen anterior to the neovagina (Fig. 1f). On MRI, heterogeneous-signal soft tissue will be seen and should not be confused for malignancy. This can be identified as the native urethra courses inside the gland along its expected anatomic course. The prostate can develop benign prostate hyperplasia and can even develop a prostatic malignancy. General guidelines regarding a prostate examination and prostate-specific antigen (PSA) testing in the age-appropriate patients should continue per guidelines in cis-male patients. A neovaginal exam can help determine prostate nodularity and suspicious nodules should be initially evaluated with transvaginal or transrectal ultrasound, being careful to understand the postoperative anatomy. As in natal males, prostate MRI can be pursued for further workup of malignancy [3].

## Conclusion

In conclusion, urologists, gynecologists, and radiologists are likely to encounter patients who have undergone gender affirmation surgeries with an increasing prevalence of these surgeries and expanding insurance coverage for the same. Knowledge of the exact surgery performed and the surgical components pursued by the patient is helpful in imaging interpretation. Being aware of the imaging findings in the immediate postoperative period along with frequently observed acute and remote complications will help physicians provide appropriate care to these patients.
